# Fructose Induces Fluconazole Resistance in *Candida albicans* through Activation of Mdr1 and Cdr1 Transporters

**DOI:** 10.3390/ijms22042127

**Published:** 2021-02-21

**Authors:** Jakub Suchodolski, Anna Krasowska

**Affiliations:** Department of Biotransformation, Faculty of Biotechnology, University of Wrocław, 50-383 Wrocław, Poland; anna.krasowska@uwr.edu.pl

**Keywords:** *Candida albicans*, fructose, diabetics, fluconazole, multidrug resistance, Mdr1p, Cdr1p

## Abstract

*Candida albicans* is a pathogenic fungus that is increasingly developing multidrug resistance (MDR), including resistance to azole drugs such as fluconazole (FLC). This is partially a result of the increased synthesis of membrane efflux transporters Cdr1p, Cdr2p, and Mdr1p. Although all these proteins can export FLC, only Cdr1p is expressed constitutively. In this study, the effect of elevated fructose, as a carbon source, on the MDR was evaluated. It was shown that fructose, elevated in the serum of diabetics, promotes FLC resistance. Using *C. albicans* strains with green fluorescent protein (GFP) tagged MDR transporters, it was determined that the FLC-resistance phenotype occurs as a result of Mdr1p activation and via the increased induction of higher Cdr1p levels. It was observed that fructose-grown *C. albicans* cells displayed a high efflux activity of both transporters as opposed to glucose-grown cells, which synthesize Cdr1p but not Mdr1p. Additionally, it was concluded that elevated fructose serum levels induce the de novo production of Mdr1p after 60 min. In combination with glucose, however, fructose induces Mdr1p production as soon as after 30 min. It is proposed that fructose may be one of the biochemical factors responsible for Mdr1p production in *C. albicans* cells.

## 1. Introduction

*Candida albicans* is an opportunistic fungal pathogen responsible for high morbidity and mortality in immunocompromised patients [1]. The most common class of antifungal drugs used for the treatment of candidiasis are azoles, which inhibit ergosterol biosynthesis by targeting cytochrome P-450 lanosterol 14α-demethylase (CYP51A1 and Erg11p) [2]. However, infections caused by *C. albicans* are recurrent and difficult to treat due to the ability of fungal cells to acquire a multidrug-resistant (MDR) phenotype [3]. In 2019, it was estimated that up to 20% of clinical *Candida* spp. isolates exhibit azole resistance [4].

One of the underlying processes responsible for MDR in *C. albicans* is the increased synthesis of membrane efflux transporters such as Cdr1p and Cdr2p, which belong to the ATP-Binding Cassette (ABC) family, and Mdr1p, which belongs to the Major Facilitator Superfamily (MFS) [5]. Although each transporter possesses different substrate specificities, all three can export fluconazole (FLC), the most common therapeutic anticandidal azole [5].

Candidiasis is associated with several diseases, including diabetes [6]. Nearly 50% of all *Candida* spp. have been identified in oral cavity samples from prediabetic patients [7]. It has been estimated that 80–90% of people with type I diabetes are carriers of *Candida* spp., and 70% of them are likely to develop infections of the skin and mucous membranes [8]. Type II diabetics are less likely to experience oral and mucosal *Candida* colonization but 10-fold more susceptible to inner organ infections [8,9]. The risk of *C. albicans* infection is increased in diabetic patients, partially due to increased serum glucose levels [8]. Glucose has already been described to promote the growth of *C. albicans*, as well as the Hog1-mediated resistance to oxidative and cationic stresses and increased resistance towards antifungal drugs [10,11,12]. We previously reported that glucose induces *CDR1* gene expression in a *C. albicans* parental strain and induces the de novo synthesis of Cdr1p in a *C. albicans cdr2Δ* mutant [13].

Recent studies have reported an increase in fructose concentration in the serum of diabetes patients [14,15], as well as accompanying several oncological diseases [16]. Fructose overabsorption is a known etiological factor of diabetes mellitus type II or metabolic syndrome [17] and is reported to promote germ tube formation and the adherence of *C. albicans* to epithelial mucous surfaces [18]. However, to date, no investigations concerning the influence of fructose on either the antifungal resistance or MDR transporter activity in *C. albicans* have been reported.

In this study, we postulate that fructose promotes a FLC resistance in *C. albicans* due to the activation of Cdr1p and by inducing the de novo synthesis of Mdr1p. We propose that elevated serum fructose levels may be one of the factors responsible for Mdr1p production in *C. albicans* cells.

## 2. Results and Discussion

### 2.1. Fructose as a Carbon Source Promotes FLC Resistance and Increased Efflux Activities of Cdr1p and Mdr1p

As a Crabtree microorganism, *C. albicans* assimilates different carbon sources at the same time, which is an adaptation to host niches where the nutrient availability may vary [19,20,21]. Fluctuations in the availability of various carbon sources have a profound effect on the physiology of *C. albicans*, including changes in the gene expression, which may result in drug resistance [22]. Here, we aimed to evaluate the effect of fructose, a carbon source naturally occurring in the human oral cavity, intestines, and blood plasma [15,16,17,19], on the sensitivity of *C. albicans* to FLC.

**Figure 1:**
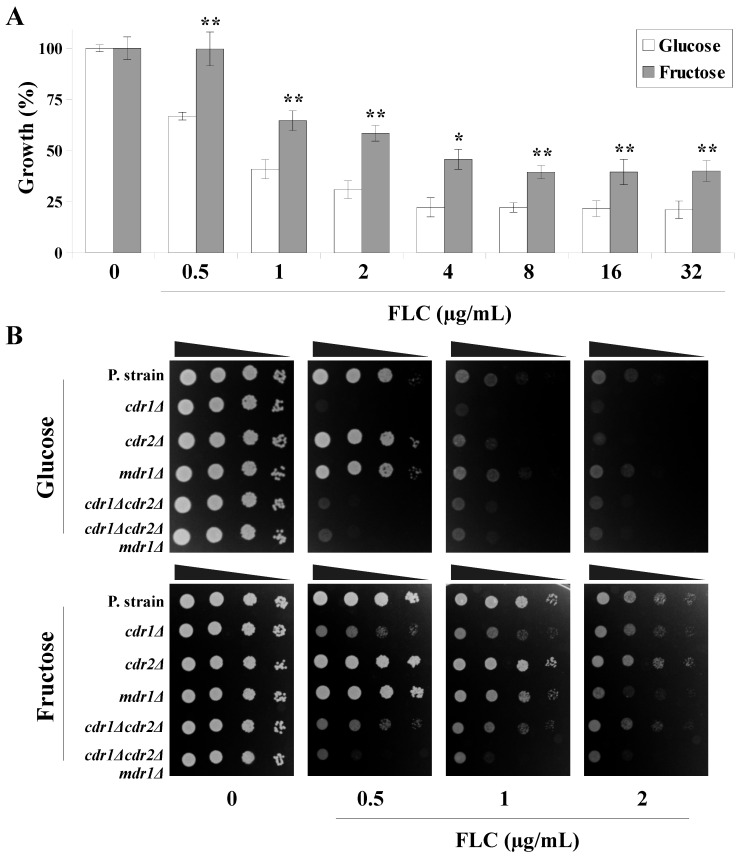
(**A**) Percentage of *Candida albicans* CAF2-1 cell growth in the presence of a range of fluconazole concentrations (FLC, 0–32 μg/mL). Cells were cultured with either yeast nitrogen base glucose (YNBG) or yeast nitrogen base fructose (YNBF) media for 24 h at 28 °C (mean ± SD, *n* = 3). Statistical analysis was performed by comparing the percentage growth between YNBG- and YNBF-grown cells at the same FLC concentrations (*, *p* < 0.05 and **, *p* < 0.01). (**B**) Growth phenotypes of *C. albicans* CAF2-1 (parental strain), DSY448 (*cdr1Δ*), DSY653 (*cdr2Δ*), DSY465 (*mdr1Δ*), DSY654 (*cdr1Δcdr2Δ*), and DSY1050 (*cdr1Δcdr2Δmdr1Δ*) strains after 48-h incubation at 28 °C. All strains were grown on either YNBG or YNBF media in the presence of a range of FLC concentrations (0–2 μg/mL).

Firstly, the growth of the *C. albicans* CAF2-1 strain in the presence of different concentrations of FLC in the YNB-based media containing glucose or fructose as a sole carbon source (Figure 1A). The growth of glucose-grown cells was inhibited ≥50% in the presence of 1-µg/mL FLC. Fructose-grown cells exhibited higher FLC tolerance, and ≥50% growth inhibition was observed only at a 4-µg/mL FLC concentration. At 8-µg/mL FLC and above, the growth of the glucose-grown cells was inhibited 80%, whereas the growth of the fructose-grown cells was inhibited only 60% (Figure 1A).

The activity of the MDR transporters is a primary factor contributing to the increased FLC tolerance among *C. albicans* isolates [23]. In order to determine the role of MDR transporters in the fructose-induced FLC resistance, we analyzed the growth phenotype of a set of isogenic *C. albicans* strains lacking one or more of the MDR transporters (Figure 1B). Clinical *C. albicans* strains isolated from patients treated with FLC display a high expression of all three transporters [24]; however, using standard laboratory conditions (general media and *C. albicans* reference strains), no gene expression or production of Cdr2p or Mdr1p are detectable [24].

In the YNB media containing glucose, the growth of parental *C. albicans* CAF2-1 cells was partially inhibited at 1-µg/mL FLC. Increasing the FLC concentration to 2 µg/mL further intensified this inhibition. A similar growth phenotype to that of CAF2-1 was observed in the DSY653 (*cdr2Δ*) and DSY465 (*mdr1Δ*) strains. However, only the residual growth of DSY448 (*cdr1Δ*), DSY654 (*cdr1Δcdr2Δ*), and DSY1050 (*cdr1Δcdr2Δmdr1Δ*) was observed, regardless of the FLC concentration used. This suggests that, in glucose-grown *C. albicans* cells, Cdr1p is primarily responsible for the FLC tolerance [25,26]. This is in agreement with previously published data, where deletion of the *CDR1* gene vastly sensitized *C. albicans* towards FLC [26,27]. Under the same conditions, the deletion of *CDR2* or *MDR1* did not influence the FLC tolerance in *C. albicans* using glucose-based media [26,27]. The reason was most likely related to the fact that the gene promoters of *CDR2* and *MDR1* are lacking a basal expression element (BEE), which is only present within the gene promoter of *CDR1* [28,29], while the synthesis of Cdr2p and Mdr1p is only induced by external factors such as azoles, fluphenazine, and β-estradiol in the case of Cdr2p or benomyl and H_2_O_2_ in the case of Mdr1p [28,29,30]. It has been reported that *MDR1* expression is not directly induced by FLC [31]; thus, Mdr1p overproduction in FLC-resistant *C. albicans* strains might be mediated by as-yet-unknown factors.

We observed that FLC at 0.5 or 1 µg/mL did not influence the growth of the parental CAF2-1, DSY653 (*cdr2Δ*), and DSY465 (*mdr1Δ*) strains grown in the medium containing fructose (Figure 1B). The DSY448 (*cdr1Δ*) and DSY654 (*cdr1Δcdr2Δ*) strains were partially inhibited; however, only the DSY1050 (*cdr1Δcdr2Δmdr1Δ*) strain exhibited an almost complete growth inhibition at all FLC concentrations used. Increasing the FLC concentration to 2 µg/mL resulted in a growth inhibition in the DSY465 (*mdr1Δ*) strain but not in the parental CAF2-1, DSY448 (*cdr1Δ*), DSY653 (*cdr2Δ*), and DSY654 (*cdr1Δcdr2Δ*) strains. We therefore concluded that fructose-grown *C. albicans* cells are tolerant towards higher FLC concentrations, most likely due to the activation of Mdr1p, whereas, at lower FLC concentrations, an additional role of either Cdr1p or Cdr2p may be responsible.

**Figure 2:**
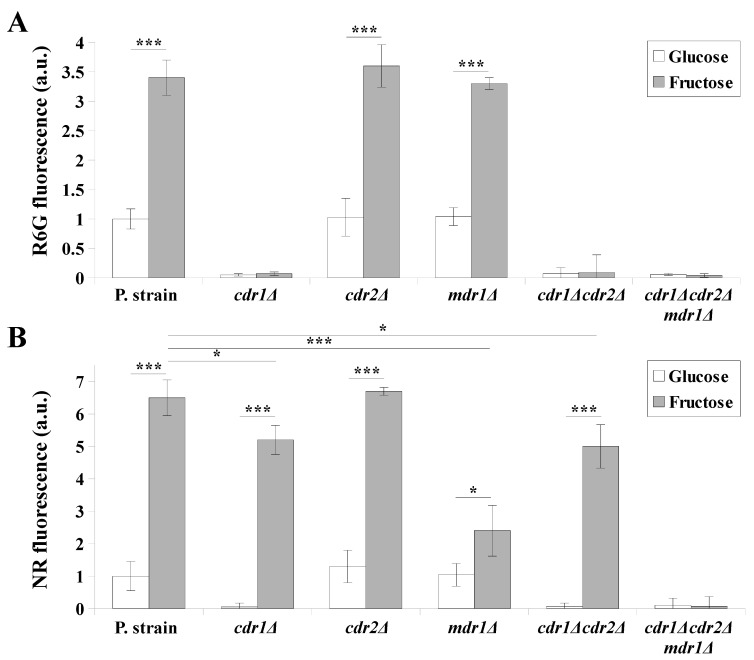
The activity of multiple drug resistance (MDR) transporters measured by (**A)** rhodamine 6G (R6G), or (**B)** Nile red (NR), efflux in *C. albicans* CAF2-1 (parental strain), DSY448 (*cdr1Δ*), DSY653 (*cdr2Δ*), DSY465 (*mdr1Δ*), DSY654 (*cdr1Δcdr2Δ*), and DSY1050 (*cdr1Δcdr2Δmdr1Δ*) strains grown in yeast nitrogen base glucose (YNBG) or yeast nitrogen base fructose (YNBF) media for 8 h at 28 °C. Fluorescence intensities (IFs) of extracellular dyes were normalized (=1 for the YNBG-grown CAF2-1 strain) (means ± SD, *n* = 3, a.u. – arbitrary units). Statistical analysis was performed between YNBG- and YNBF-grown cells or between different strains (*, *p* < 0.05, and ***, *p* < 0.001).

To confirm these conclusions, we evaluated the efflux activities of MDR pumps using the same set of *C. albicans* strains grown in either glucose- or fructose-containing media (Figure 2). For this purpose, we used two fluorescent dyes: rhodamine 6G (R6G), which is a substrate of Cdr1p and Cdr2p but not Mdr1p, and Nile red (NR), which is a substrate of Cdr1p and Mdr1p but not Cdr2p [32]. Both the fluorescent substrates accumulate within the yeast cells and are actively removed by the transporters, which is measured as an extracellular fluorescence, and reflects the efflux activity of the transporters [32].

The efflux of R6G was observed only in the case of *C. albicans* strains that contain Cdr1p (CAF2-1, DSY653, and DSY465), regardless of the carbon source used (Figure 2A). However, in these strains, the R6G efflux was ~3.5-fold higher in the media containing fructose (Figure 2A). Based on these observations, we concluded that Cdr2p is probably not activated on either glucose- or fructose-containing media, and Cdr1p activity is higher in the case of fructose-grown cells.

The efflux of NR in glucose-grown cells was observed in strains expressing Cdr1p (CAF2-1, DSY653, and DSY465) a comparable level (Figure 2B) to the efflux of R6G at the same carbon source (Figure 2A). The NR efflux in fructose-grown cells was observed in all *C. albicans* strains except for DSY1050, which is deficient in all MDR transporters. The *C. albicans* strains that contain both Cdr1p and Mdr1p (CAF2-1 and DSY653) were characterized by a ~6-fold higher NR efflux when grown on the fructose-containing medium. The strains that contain Mdr1p but not Cdr1p (DSY448 and DSY654) displayed a ~5-fold higher NR efflux when grown in the fructose-containing medium. The strain lacking Mdr1p but expressing Cdr1p (DSY465) was characterized by a ~2.5-fold higher NR efflux when grown on fructose. This suggested that fructose-grown cells, despite a higher Cdr1p-dependent efflux activity, additionally feature an active Mdr1 transporter.

### 2.2. Fructose-Grown Cells Are Characterized by High Levels of Cdr1p and Mdr1p

**Figure 3:**
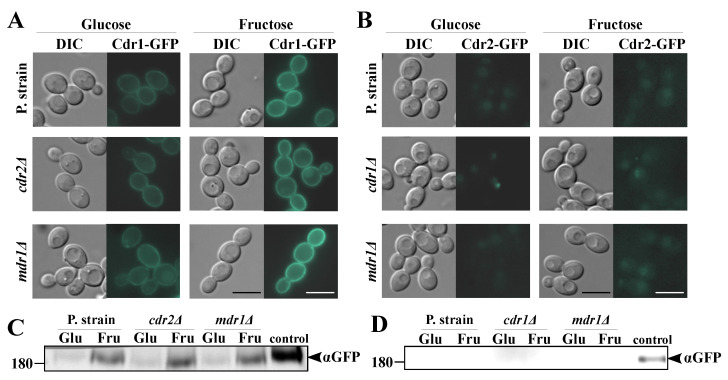
Fluorescence micrographs of the subcellular localization of the (**A**) Cdr1p-green fluorescent protein (GFP) in the *C. albicans* strains, KS052 (CAF2-1 *CDR1-GFP*), KS053 (DSY653 *CDR1-GFP*), and KS054 (DSY465 *CDR1-GFP*) or (**B**) Cdr2p-GFP in the *C. albicans* strains KS063 (CAF2-1 *CDR2-GFP*), KS064 (DSY448 *CDR2-GFP*), and KS065 (DSY465 *CDR2-GFP*). Scale bar = 5 μm. (**C**) Immunoblot analysis of Cdr1p-GFP in *C. albicans* KS052, KS053, and KS054. A positive control was prepared by treating the KS052 strain with 4-µg/mL FLC for 4 h. (**D**) Immunoblot analysis of Cdr2p-GFP in *C. albicans* KS063, KS064, and KS065. A positive control was prepared by treating the KS063 strain with 20-µg/mL fluphenazine for 4 h. In all the presented experiments, the *C. albicans* strains were grown in yeast nitrogen base glucose (YNBG) or yeast nitrogen base fructose (YNBF) media for 8 h at 28 °C.

**Figure 4:**
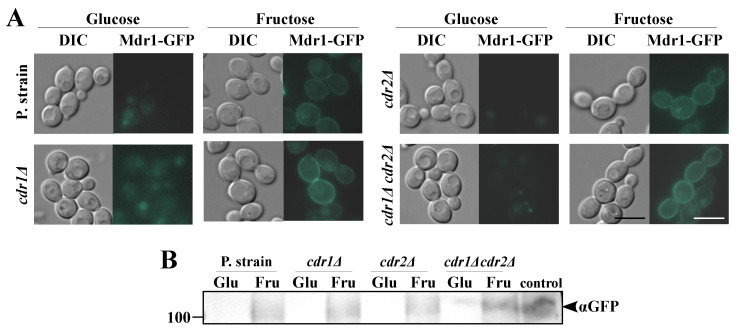
(**A**) Fluorescence micrographs of the subcellular localization of Mdr1p-GFP in the *C. albicans* strains KS070 (CAF2-1 *MDR1-GFP*), KS075 (DSY448 *MDR1-GFP*), KS074 (DSY653 *MDR1-GFP*), and KS073 (DSY654 *MDR1-GFP*). Scale bar = 5 μm. (**B**) Immunoblot analysis of Mdr1p-GFP in *C. albicans* KS070, KS075, KS074, and KS073. A positive control was obtained by treating the KS070 strain with 3% H_2_O_2_ for 4 h. In both presented experiments, the *C. albicans* strains were grown in yeast nitrogen base glucose (YNBG) or yeast nitrogen base fructose (YNBF) media for 8 h at 28 °C.

The results described in Section 2.1, together with previous reports, suggest that, in the case of glucose-grown *C. albicans* cells, Cdr1p is primarily responsible for the FLC tolerance, with negligible roles played by Cdr2p or Mdr1p [26,27]. However, in *C. albicans* cells grown on fructose, particularly in the case of strains positive for the *MDR1* gene but negative for the *CDR1* gene, we observed a high FLC tolerance (Figure 1B) and high NR efflux (Figure 2B). We concluded that these observations may result from the synthesis of Mdr1p in *C. albicans* cells as a result of the growth in the presence of fructose. In order to confirm those conclusions, we constructed a series of GFP-tagged *C. albicans* strains. We labeled Cdr1p-GFP in the *cdr2Δ* or *mdr1Δ* backgrounds; Cdr2p-GFP in the *cdr1Δ* or *mdr1Δ* backgrounds; and Mdr1p-GFP in the *cdr1Δ, cdr2Δ*, or *cdr1Δcdr2Δ* backgrounds (Table 1). We performed microscopic observations of the fluorescent signal in the constructed *C. albicans* strains grown in either glucose or fructose media, which were further validated by Western blotting (Figure 3 and Figure 4).

The Cdr1p-GFP signal was observed in the plasma membranes of *C. albicans* in all the aforementioned conditions (Figure 3A). However, the Cdr1p-GFP signal was visibly stronger in fructose-grown cells than in glucose-grown cells. A Western blotting protein analysis (Figure 3C) confirmed those observations (Figure 3C). Additionally, it was concluded that the absence of detectable Cdr2p or Mdr1p does not influence the level of Cdr1p (Figure 3A). In contrast, we previously observed that the presence of glucose increased the Cdr1p levels in the *C. albicans cdr2Δ* strain to a greater extent than in the parental strain [13]. It must be noted that, previously, we performed only short-term glucose induction (12 or 36 min). It may therefore be concluded that short-time exposure of the *C. albicans cdr2Δ* strain to glucose results an increased production of Cdr1p, while, after long-term incubation with glucose, the Cdr1p protein level eventually stabilizes.

The Cdr2p-GFP signal was not detected under either of the experimental conditions (Figure 3B,D). This shows that, regardless of the carbon source (glucose or fructose), Cdr2p was not involved in either the FLC resistance (Figure 1) or R6G efflux (Figure 2A). Additionally, this explains the lack of differences in R6G efflux between the parental CAF2-1 and DSY653 (*cdr2Δ*) strains (Figure 2A). Additionally, we observed that the absence of Cdr1p or Mdr1p does not induce the production of Cdr2p. Kolaczkowska et al. [33] previously reported that, upon disruption of the *PDR5* gene, which encodes an ABC transporter homologous of Cdr1p in *Saccharomyces cerevisiae,* a compensatory activation of other ABC transporters (Snq2 and Yor1) occurs. Similar observations were reported for ABC transporters of pathogenic fungus *Trichophyton* spp. [34]. In *C. albicans*, the transcriptional regulation of the *CDR1* and *CDR2* genes overlaps: Mrr2, Upc2, Ndt80, and Znc1 act as positive regulators [35,36], and Flo8 as a negative regulator [36] of both genes. However, our results suggest that the disruption of either *C. albicans* ABC transporters does not induce the production of the remaining protein (Figure 3).

Cells cultured in a medium with glucose, in contrast to those cultured with fructose, did not synthesize Mdr1p, which was the reason for the absence of the Mdr1p-GFP signal during the microscopic observations and the lack of Mdr1p detected by Western blot (Figure 4). Moreover, in the *C. albicans* KS073 strain, which lacks both the Cdr1 and Cdr2 proteins, we observed a more pronounced Mdr1p-GFP signal. Based on these observations, we concluded that *C. albicans* cells synthesize Mdr1p in the presence of fructose without any other stimulating factor (Figure 4B).

The promoter of the *MDR1* gene includes a H_2_O_2_ responsive element (HRE), which induces the production of Mdr1p upon oxidative stress [37], which led us to hypothesize that the fructose metabolism might induce oxidative stress. Conversely, however, fructose has been described to exhibit a general protective effect against oxidative stress in *S. cerevisiae* cells [38], including a specific protection against H_2_O_2_ and reactive oxygen species (ROS) [39]. This led us to a different hypothesis. In eukaryotic cells, fructose, like glucose, is metabolized to pyruvate, which supplies energy to cells through the Krebs cycle [40]. However, fructose has been described to also be metabolized to a toxic glycolytic byproduct called methylglyoxal (MG), the elevated levels of which are responsible for hepatotoxicity in diabetic patients [40]. In *Candida lusitaniae*, the MG metabolism is believed to be mediated by Mgd1 and Mgd2 reductases, the expression of which is controlled by Mrr1p, which, in turn, is inducible by MG [41]. The homologous protein in *C. albicans* (*Ca*Mrr1p) is a major transcriptional inductor of *MDR1* [42]. Thus, we hypothesize that the fructose metabolism, leading to the production of MG, might directly induce expression of the *MDR1* gene and production of Mdr1p through the activation of Mrr1p.

### 2.3. Serum Levels of Fructose Induces de Novo Synthesis of Mdr1p and Enhanced Synthesis of Cdr1p

The concentrations of glucose and fructose in different niches of the human body—specifically, the digestive tract and bloodstream—depend mostly on dietary factors. The ingestion of sugar-rich products may lead to an increase in fructose concentration in the peripheral venous blood of up to ~0.006%, and the glucose concentration of up to ~0.2% [19,43]. Rodaki et al. [10] reported that a short-term exposure of *C. albicans* to 0.1% glucose induces a stress response, which includes the transcriptional activation of *CDR1*.

**Figure 5:**
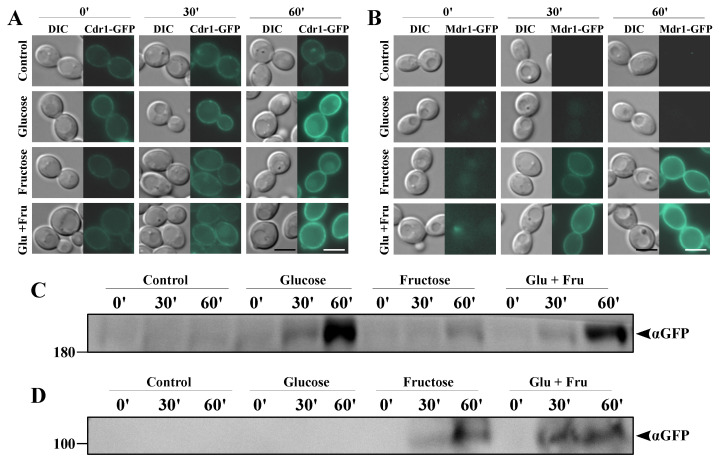
Fluorescence micrographs showing the subcellular localization of (**A**) Cdr1p-GFP in the *C. albicans* strain KS052 (CAF2-1 *CDR1-GFP*) or (**B**) Mdr1p-GFP in the *C. albicans* strain KS070 (CAF2-1 *MDR1-GFP*). Scale bar = 3 μm. Immunoblot analysis of (**C**) Cdr1p-GFP in *C. albicans* KS052 or (**D**) Mdr1-GFP in *C. albicans* KS070. In all the presented experiments, the *C. albicans* strains were grown in a yeast nitrogen base glucose (YNBG) medium for 8 h at 28 °C; starved in a HEPES-NaOH buffer for 1 h at 28 °C; and induced with 0.2% glucose, 0.006% fructose, or both sugars simultaneously (Glu + Fru) for 30 or 60 min.

We report that *C. albicans* cells, grown up until the early logarithmic phase with fructose as the sole carbon source, are characterized by the presence of Mdr1p (Figure 4) and increased levels of Cdr1p (Figure 3). We aimed to investigate whether this effect occurs upon the short-term exposure of *C. albicans* to glucose, fructose, or both sugars in the bloodstream. To this end, we cultured the *C. albicans* KS052 (Cdr1p-GFP) and KS070 (Mdr1p-GFP) strains in YNBG medium until the early logarithmic phase; at which point, we induced cell starvation by incubating cells for one hour in a HEPES-NaOH buffer. The starved cells were supplemented with either glucose (0.2%), fructose (0.006%), or both sugars and analyzed for the expression of the Cdr1 and Mdr1 proteins (Figure 5).

We observed that *C. albicans* KS052 cells exposed to glucose or a glucose–fructose mixture are characterized by pronounced Cdr1p-GFP fluorescence in the plasma membrane (Figure 5A). Western blotting revealed an increasing Cdr1p-GFP signal proportionate to the increase in incubation time with glucose or a glucose–fructose mixture (Figure 5C). This suggests an increase in Cdr1p synthesis induced by glucose, which is in agreement with the data reported by Rodaki et al. [10] and Szczepaniak et al. [13]. However, we observed a slightly more pronounced fluorescence of Cdr1p-GFP in cells exposed to fructose, as well as a slightly increased signal seen with Western blotting (Figure 5).

We observed no Mdr1p-GFP signal at the beginning of the induction (time = 0 min), which is in agreement with the data presented in Figure 4. The exposure of the *C. albicans* KS070 strain to glucose did not lead to the synthesis of Mdr1p-GFP. Only exposure to fructose induced a detectable Mdr1p-GFP signal (Figure 5). Thus, it may be concluded that the exposure of *C. albicans* to low concentrations of fructose induces a de novo synthesis of Mdr1p after only 30 min of exposure.

We conclude that fructose as a carbon source induces FLC resistance in *C. albicans* in laboratory conditions in a general culture media. However, our observations may be of particular importance, as enhanced concentrations of fructose in the bloodstream persist for a much longer time than glucose (~three hours after fructose ingestion) before returning to the baseline levels [43].

## 3. Conclusions

These findings demonstrate that fructose as a carbon source enhances the FLC resistance in *Candida albicans* by two modes: the activation of Mdr1p and by inducing elevated levels of Cdr1p. We observed that fructose-grown *C. albicans* cells have a higher efflux activity of both transporters as opposed to glucose-grown cells, which constitutively synthesize only Cdr1p. Additionally, we concluded that the fructose serum level of 0.006% induces the de novo production of Mdr1p.

## 4. Materials and Methods

### 4.1. Chemicals

Chemicals and reagents used in this study were purchased from the following sources: sodium dodecyl sulfate (SDS), 2-deoxy-d-glucose, fluconazole (FLC), rhodamine 6G (R6G), Nile red (NR), and lithium acetate (LiAc) (Sigma-Aldrich, Poznań, Poland); commercial antibodies: mouse monoclonal anti-green fluorescent protein (αGFP) (manufacturer: Roche and distributor: Sigma-Aldrich, Poznań, Poland) and horseradish peroxidase (HRP) conjugated rabbit anti-mouse (manufacturer: GE Healthcare and distributor: Sigma-Aldrich, Poznań, Poland); d-glucose, d-fructose, bacteriological agar, HEPES, Tris, and EDTA (manufacturer: Bioshop and distributor: Lab Empire, Rzeszów, Poland); yeast nitrogen base (YNB), yeast extract (YE), peptone, and sorbitol (manufacturer: BD and distributor: Diag-med, Warszawa, Poland); nourseothricin (NAT) (Jena Bioscience, Jena, Germany); and dithiothreitol (DTT) (A&A Biotechnology, Gdynia, Poland). All chemicals were high-purity grade.

### 4.2. Strains and Growth Conditions

The *C. albicans* strains used in this study are listed in Table 1. CAF2-1, DSY448, DSY653, DSY465, DSY654, and DSY1050 were kind gifts from Professor D. Sanglard (Lausanne, Switzerland). KS052 and KS068 were previously constructed by our group, while KS053, KS054, KS064, KS065, KS070, KS073, KS074, and KS075 were constructed for the purposes of this study. Strains were pregrown at 28 °C on yeast nitrogen base glucose (YNBG) or yeast nitrogen base fructose (YNBF) media (0.67% YNB containing 2% glucose or 2% fructose, respectively) in an incubator while shaking at 120 rpm. Agar was added at a final concentration of 2% for medium solidification.

For most of the experiments, cells were grown until they reached the early logarithmic phase (8 h). Growth phases were determined as previously described [44]. Cells were centrifuged at 4500 rcf (relative centrifugal force) for 5 min; washed twice (4500 rcf, 5 min) with either phosphate-buffered saline (PBS), H_2_O_dd_, or 50-mM HEPES–NaOH buffer (pH 7.0); and resuspended in either PBS, H_2_O_dd_, or HEPES-NaOH to the indicated A_600_.

For the induction experiments, *C. albicans* suspensions in HEPES-NaOH (A_600_ = 1.0 in 25 mL) were incubated for 60 min at 28 °C. Subsequently, the cells were centrifuged at 4500 rcf for 5 min, washed twice (4500 rcf, 5 min) with HEPES-NaOH, and resuspended in HEPES-NaOH. Lastly, the cells were treated with glucose (0.2%), fructose (0.006%), or both sugars for either 30 or 60 min.

### 4.3. Plasmids and Strains Construction

Plasmid pGFP-NAT1 [47] was a generous gift from Professor S. Bates (Exeter, United Kingdom). The CDR1-GFP-NAT1 and CDR2-GFP-NAT1 cassettes were prepared as described previously [46]. Briefly, both cassettes were amplified from pGFP-NAT1 using the primer pairs C1_GFPNAT_F and C1_GFPNAT_R or C2_GFPNAT_F and C2_GFPNAT_R. The MDR1-GFP-NAT1 cassette was amplified from pGFP-NAT1 using the primer pair M1_GFPNAT_F and M1_GFPNAT_R.

*C. albicans* strains were transformed by electroporation with the linear gel-purified *CDR1-GFP-NAT1*, *CDR2-GFP-NAT1*, or *MDR1-GFP-NAT1* cassettes according to the protocols described by Sasse et al. [48] with modifications. Briefly, *C. albicans* cells were cultured in YPD medium (1% YE, 1% peptone, and 2% glucose) until they reached the early stationary growth phase (16 h). Cells were then centrifuged at 4500 rcf for 5 min; washed (4500 rcf, 5 min) with H_2_O_dd_; resuspended in TE-LiAc buffer (10-mM Tris-HCl, 1-mM EDTA, and 0.1-M LiAc, pH 8); and incubated at 28 °C for 60 min with shaking at 120 rpm. Subsequently, 0.025-M DTT was added for further incubation (28 °C, 30 min, and shaking at 120 rpm). The cells were again centrifuged at 4500 rcf for 5 min; washed twice (4500 rcf, 5 min, and 4 °C) with ice-cold H_2_O_dd_; washed (4500 rcf, 5 min, and 4 °C) with ice-cold 1-M sorbitol; and concentrated in ice-cold 1-M sorbitol. Electrocompetent cells were transformed (1.8 kV, 200 Ω, and 25 uF) using a Gene Pulser II electroporator (Bio-Rad, Warsaw, Poland), washed (2500 rcf, 5 min) with 1=M sorbitol, resuspended in YPD, and incubated at 28 °C for 4 h with shaking at 120 rpm. Finally, transformed cells were selected on YPD using 200-µg/mL NAT.

The presence of the *NAT1* marker was verified using the primer pair NAT1_F and NAT1_R. The correct integration of the cassette into the genomic locus was verified using the primer pairs C1NAT1_SF and GFP_N1_SR2 (for the *CDR1/CDR1-GFP-NAT1* strains), C2NAT1_SF and GFP_N1_SR2 (for the *CDR1/CDR1-GFP-NAT1* strains), or M1NAT1_SF and GFP-N1-SR2 (for the *MDR1/MDR1-GFP-NAT1* strains). All the primers sequences are detailed in Table 2.

### 4.4. Percentage of Growth

To assess the effects of FLC on *C. albicans* growth in the presence of different carbon sources, we followed the protocol described by the Clinical and Laboratory Standards Institute (2008), 3rd ed. M27-A3 [49] with modifications. Briefly, 10-mg/mL stock solution of FLC was serially diluted in YNBG or YNBF media using 96-well sterile plates (Sarstedt, Nümbrecht, Germany). The various media compositions were then inoculated with *C. albicans* suspensions (final A_600_ = 0.01 per well) and prepared in fresh YNBG or YNBF media from 24-h YNBG cultures. After 24 h of incubation at 28 °C, A_600_ was measured using a ASYS UVM 340 microplate reader (Biogenet, Józefów, Poland). The percentage of growth of the *C. albicans* CAF2-1, DSY448, DSY653, DSY465, DSY654, and DSY1050 strains was determined by normalizing A_600_ to that observed under conditions without FLC.

### 4.5. Phenotypic Tests

PBS suspensions of *C. albicans* CAF2-1, DSY448, DSY653, DSY465, DSY654, and DSY1050 (A_600_ = 0.7), prepared from overnight YNBG cultures, were serially diluted with PBS in a range of 10^0^ to 10^−3^. Next, 3 µL of each dilution were spotted onto either YNBG- or YNBF-based agar containing FLC (0.5–2 µg/mL). After cultivation for 48 h at 28 °C, the plates were photographed using a FastGene^®^ B/G GelPic imaging box (Nippon Genetics, purchased from Abo, Gdańsk, Poland).

### 4.6. Efflux Activity of MDR Transporters

The efflux assay was performed according to the protocol of Szczepaniak et al. [50] with modifications. Briefly, 25-mL *C. albicans* suspensions (A_600_ = 1.0 in 25-mL HEPES-NaOH) were treated with 5-mM 2-deoxy-D-glucose and incubated at 28 °C for 60 min with shaking at 200 rpm. Subsequently, 10-μM R6G or 7-μM NR were added before further incubation at 28 °C for 90 min with shaking at 200 rpm. Following this, the cells were centrifuged at 4500 rcf for 5 min, washed twice (4500 rcf, 5 min) with HEPES-NaOH, concentrated to 2 mL in HEPES-NaOH (A_600_ = 10), and incubated at 28 °C for 5 min with shaking at 200 rpm. For each condition, the dye uptake was always ≥95%. Intensities of fluorescence (FIs) were measured 30 min after efflux. The assay was performed using a Cary Eclipse spectrofluorometer (Agilent Technologies, Santa Clara, CA, USA). The probes were excited at 529 nm (Ex slit = 5 nm), and emission was recorded at 553 nm (Em slit = 10 nm). IFs were normalized to 1 for the efflux activity of the control conditions (parental strain grown in YNBG).

### 4.7. Microscopic Studies

The *CDR1-GFP*, *CDR2-GFP*, or *MDR1-GFP* strains were suspended in PBS, concentrated, and observed under a Zeiss Axio Imager A2 microscope equipped with a Zeiss Axiocam 503 mono microscope camera and a Zeiss HBO100 mercury lamp (Zeiss, Poznań, Poland).

### 4.8. Western Blotting

Crude protein extracts from *CDR1-GFP*, *CDR2-GFP*, or *MDR1-GFP* strains were isolated as previously described [44,50]. Electrophoretic separation and transfer of Cdr1p-GFP was performed as previously described [50]. For Mdr1p-GFP separation, the following modification was applied: crude proteins from *MDR1-GFP* strains were separated on 8% SDS-polyacrylamide gels. For detection, mouse αGFP primary antibodies were used, followed by HRP-conjugated rabbit anti-mouse secondary antibodies. The remaining steps were performed as described in Reference [50].

### 4.9. Statistical Analysis

Unless stated otherwise, data represent the means ± standard errors from at least 3 biological replicates. Microscopic observations and Western blot analyses were performed at least in 2 independent replicates, of which the representatives were included in the figures. Statistical significance was determined using a Student’s *t*-test (binomial, unpaired).

## Figures and Tables

**Table 1:** *Candida albicans* Strains Used in This Study.

Strain	Parent	Description	Complete Genotype	Reference
CAF2-1		Parental strain	*ura3∆::imm434/URA3*	[45]
DSY448	CAF2-1	*cdr1Δ*	*ura3Δ::imm434/ura3Δ::imm434* *cdr1Δ::hisG/cdr1Δ::hisG-URA3-hisG*	[26]
DSY653	CAF2-1	*cdr2Δ*	*ura3Δ::imm434/ura3Δ::imm434* *cdr2Δ::hisG/cdr2Δ::hisG-URA3-hisG*	[25]
DSY465	CAF2-1	*mdr1Δ*	*ura3Δ::imm434/ura3Δ::imm434* *mdr1Δ::hisG/mdr1Δ::hisG-URA3-hisG*	[26]
DSY654	CAF2-1	*cdr1Δcdr2Δ*	*ura3Δ::imm434/ura3Δ::imm434* *cdr1Δ::hisG/cdr1Δ::hisG* *cdr2Δ::hisG/cdr2Δ::hisG-URA3-hisG*	[25]
DSY1050	CAF2-1	*cdr1Δcdr2Δmdr1Δ*	*ura3Δ::imm434/ura3Δ::imm434* *cdr1Δ::hisG/cdr1Δ::hisG* *cdr2Δ::hisG/cdr2Δ:: hisG* *mdr1Δ::hisG/mdr1Δ::hisG-URA3-hisG*	[27]
KS052	CAF2-1	*CDR1-GFP*	*ura3Δ::imm434/URA3 CDR1/CDR1-GFP-NAT1*	[46]
KS053	DSY653	*cdr2Δ CDR1-GFP*	*ura3Δ::imm434/ura3Δ::imm434* *cdr2Δ::hisG/cdr2Δ::hisG-URA3-hisG* *CDR1/CDR1-GFP-NAT1*	This study
KS054	DSY465	*mdr1Δ CDR1-GFP*	*ura3Δ::imm434/ura3Δ::imm434* *mdr1Δ::hisG/mdr1Δ::hisG-URA3-hisG* *CDR1/CDR1-GFP-NAT1*	This study
KS063	CAF2-1	*CDR2-GFP*	*ura3Δ::imm434/URA3 CDR2/CDR2-GFP-NAT1*	[46]
KS064	DSY448	*cdr1Δ CDR2-GFP*	*ura3Δ::imm434/ura3Δ::imm434* *cdr1Δ::hisG/cdr1Δ::hisG-URA3-hisG* *CDR2/CDR2-GFP-NAT1*	This study
KS065	DSY465	*mdr1Δ CDR2-GFP*	*ura3Δ::imm434/ura3Δ::imm434* *mdr1Δ::hisG/mdr1Δ::hisG-URA3-hisG* *CDR2/CDR2-GFP-NAT1*	This study
KS070	CAF2-1	*MDR1-GFP*	*ura3Δ* *::imm434/URA3 MDR1/MDR1-GFP-NAT1*	This study
KS073	DSY654	*cdr1Δcdr2Δ* *MDR1-GFP*	*ura3Δ::imm434/ura3Δ::imm434* *cdr1Δ::hisG/cdr1Δ::hisG* *cdr2Δ::hisG/cdr2Δ::hisG-URA3-hisG* *MDR1/MDR1-GFP-NAT1*	This study
KS074	DSY653	*cdr2Δ* *MDR1-GFP*	*ura3Δ::imm434/ura3Δ::imm434* *cdr2Δ::hisG/cdr2Δ::hisG-URA3-hisG* *MDR1/MDR1-GFP-NAT1*	This study
KS075	DSY448	*cdr1Δ* *MDR1-GFP*	*ura3Δ::imm434/ura3Δ::imm434* *cdr1Δ::hisG/cdr1Δ::hisG-URA3-hisG* *MDR1/MDR1-GFP-NAT1*	This study

**Table 2:** Primers Used in This Study.

Primer	Sequence 5′–3′
C1_GFPNAT_F	CATTCTTACGGTGATCTTTTATTGGTTAGCTAGAGTTCCAAAGGGTAACA GAGAGAAAAAAAATAAGAAAGGTGGTGGTTCTAAAGGTGAAGAATTATT
C1_GFPNAT_R	AACAACAACAATAGTCTAAAAACGTCTATTATATTTTAGACGTTTGAGA TACCACCATGTCAAAAAACAACGTTAGTATCGAATCG ACAGC
C2_GFPNAT_F	CATTCTTACTATTTTCTTTTACTGGTTGGCTAGAGTTCCAAAAGGTAATAG AGAAAAGAAGATGAAAAAAGGTGGTGGTTCTAAAG GTGAAGAATTATT
C2_GFPNAT_R	ATCAAACAATCACAAATAACGTATAAATAATAATAAGAAAAAAAAAAT ATGAATACTAATTGTAAAATAACGTTAGTATCGAATCG ACAGC
M1_GFPNAT_F	TTGTTATGATTGCTATTCCAGTTTTGTTTTACTTGAACGGACCAAAGTTGA GAGCAAGATCTAAGTATGCGGTGGTGGTTCTAAAGGTGAAGAATTATT
M1_GFPNAT_R	TCAGTCCTTTTCTCTTTTTAATTATTGATTAATGTATCTATAACACGATATA TCTATAGGAAAACAATGACGTTAGTATCGAATCGACAGC
NAT1_F	GCTTATAGATACAGAACTTCTGTTCC
NAT1_R	TGAAACCCATTCTTCTATAAGCATG
C1NAT1_SF	TCAAGCTATGCTTTCTACTGGA
C2NAT1_SF	GTATTGGCTGGTCCTAATGTG
M1NAT1_SF	TATTGGTATTGTCATTGCTGCC
GFP_N1_SR2	AATTCTTCACCTTTAGAACCACC

## Data Availability

The data presented in this study are available on request from the corresponding author (J.S.).
